# Understanding how behaviour therapists use autism spectrum disorder diagnostic information for intervention planning

**DOI:** 10.3389/fpsyt.2023.1242748

**Published:** 2023-09-25

**Authors:** Isabelle Caven, Claire Nguyen, Justine Wiegelmann, Erica Laframboise, Melanie Penner

**Affiliations:** ^1^Holland Bloorview Kids Rehabilitation Hospital, Toronto, ON, Canada; ^2^Department of Paediatrics, Michael Garron Hospital, Toronto, ON, Canada; ^3^Department of Paediatrics, McMaster University, Hamilton, ON, Canada

**Keywords:** autism, intervention, behaviour therapists, applied behaviour analysis, early intensive behaviour intervention

## Abstract

Understanding how behaviour therapists incorporate diagnostic assessments into their intervention planning can help to streamline assessment procedures and facilitate communication. The objectives are to identify what information from the diagnostic assessment is received by behaviour therapists and which assessment elements are most important and relevant for treatment planning. Behaviour therapists, identified through Ontario registries, were surveyed about their use of diagnostic information in treatment planning. Seventy-one behaviour therapists completed the survey (response rate = 35.5%). The diagnostic information most frequently received by respondents included brief (69%) and detailed (49.2%) physician/psychologist report, speech/language assessment report (52.1%) and individualised education plan (50.7%). Most respondents indicated that information from the physician/psychologist report is often out-dated (74.6% Agree/Strongly Agree). There was variable agreement that the information in the diagnostic package influences the type and quantity of treatment. These findings demonstrate that while diagnostic assessments received by behaviour therapists are important to their planning, other independently obtained sources of information, such as client interviews, are relatively more important to this process. The diagnostic assessment is one tool to inform treatment planning; however, up-to-date information about the child’s needs is likely to be more informative.

## 1. Introduction

Autistic children and their families frequently access early intensive behavioural intervention (EIBI) and other applied behaviour analysis (ABA)-based therapies (1). The broad aim of ABA is to use the principles of behaviour analysis to design, implement, and evaluate modifications that produce socially significant changes in behaviour (2). The science of behaviour analysis is rooted in B.F. Skinner’s radical behaviourism (2). Recent meta-analyses have explored the efficacy of behavioural interventions. Sandbank, Bottema-Beutel (3) found that behavioural interventions have evidence to support their effectiveness; however, the strength of this evidence this is limited by issues of methodological rigour, including detection bias as a result of caregiver/teacher report (3). Other meta-analyses also identified that EIBI could improve adaptive behaviour (1, 4) and cognitive ability (1), although this evidence was also rated as weak.

Limited information about access to behavioural therapy exists for Canada. The OAP reported that, of families electronically surveyed about their choice of services, 80% indicated they would choose ABA for their child (5). In the United States, between 2018 and 2021 the number of Board Certified Behaviour Analysts (BCBAs) increased by 65% (6). Despite this increase in BCBAs, research from an American state reported the average wait time to EIBI is approximately 9 months (7).

While behaviour therapists may play a key role in the therapeutic pathways of many autistic children and their families, they are often not involved in the diagnostic assessment process. A review of the literature found no published research relating to how behaviour therapists use or incorporate diagnostic assessment information into their treatment planning. The Ontario Autism Program describes physicians, family support providers, educators and clinicians as potential sources of information for assessments that inform intervention planning or delivery (8). Numerous guidelines have stated that a multidisciplinary autism diagnostic assessment is required to inform treatment planning (9), yet no information currently exists as to *how* behaviour therapists incorporate this information into treatment planning or delivery. It is important to understand how the information obtained from a multidisciplinary assessment is used to influence treatment planning, especially considering that some have argued that due the resource intensiveness of this type of assessment, it may not be necessary in all cases (10) and could contribute to lengthy diagnostic wait times. Understanding how behaviour therapists use diagnostic information obtained from various sources (school reports, allied health assessments, physician assessments, etc.), may help to improve collaboration between diagnosticians and therapists, streamlining pathways and improving family experiences.

To address this knowledge gap, we undertook a descriptive study using a bespoke online questionnaire for behaviour therapists to address three objectives: (1) identify what information from the diagnostic assessment is received; (2) determine which elements of the diagnostic assessment are most important for treatment planning; and (3) determining the quality, quantity, and relevance of various aspects of the diagnostic assessment.

## 2. Methods

### 2.1. Participants

Public registries of Ontario-based behaviour therapists were used to identify potential participants, which was estimated to comprise around 200 behaviour therapists at time of survey dissemination. Providers of ABA-based services are generally referred to as behaviour therapists and have a range of certifications. A BCBA has a Master’s degree and 2,000 h of supervised fieldwork, and a BCBA-D is a BCBA with a Doctoral degree (11). A Board Certified Assistant Behaviour Analyst (BCaBA) has at least a Bachelor’s degree and 1,000 h of supervised practiced, and is supervised by a BCBA or BCBA-D. Lastly, Registered Behaviour Technicians (RBTs) practice under the supervision of a BCBA, BCBA-D and/or a BCaBA, and are required to have a high school diploma, 40 h of training, and to have passed a competency assessment. BCBAs, BCBA-Ds, and BCaBAs are all required to pass an exam to receive their certification (12).

The survey was completed in its entirety by 71 behaviour therapists (response rate of 35.5%) who were involved in in treatment programming/planning. Participant email addresses were from the OAP Provider Registry and/or Ontario Association for Behaviour Analysis and/or BACB Certification Board Registrants in Ontario, Canada. Potential respondents also had to actively work with autistic children in the last 2 years and have access to a computer and the ability to complete a questionnaire in English. The means and frequencies of the demographic characteristics of respondents were calculated, and presented in Table 1. Most respondents identified as female (*n* = 68, 95.8%) and had completed a master’s degree (*n* = 62, 87.3%). The mean number of months of clinical practice training was 54.9 (*SD* = 119.6), and number of years working in their role was 11.2 (*SD* = 7). The most frequently identified type of certification was BCBA (*n* = 54, 76.1%), and most identified working in a large urban population centre (more than 100,000 people).

**Table 1:** Demographic, practice, and education information of respondents (*n* = 71).

	*n*	%	Mean	Standard deviation
Age				
20–30 years	17	23.9		
31–40 years	35	49.3		
41–50 years	15	21.6		
51–60 years	4	5.6		
Gender				
Female	68	95.8		
Male	3	4.2		
Education				
Bachelor’s degree	9	12.7		
Master’s degree	62	87.3		
Behaviour therapy training (months)				
Formal education (i.e. supervised field work, degree programme or board certification)	69		45.3	36.7
Clinical practice	65		54.9	119.6
Time working as behaviour analyst (years)	71		11.2	7
Main practice setting				
For profit (group)/clinic	16	22.5		
Not-for-profit centre/clinic	15	21.1		
For profit independent	30	42.3		
Not-for-profit independent	2	2.8		
Other	8	11.3		
Type of service delivery				
One-to-one	36	50.7		
Mediator model/consultation	24	33.8		
Parent training/workshop	3	4.2		
Group	1	1.4		
Other	7	9.9		
Age of clients^a^				
Preschool	52	73.2		
Kindergarten to grade 8	66	92.9		
High school	43	60.6		
Geographical location of practice				
Large urban population centre (>100,000 people)	53	74.6		
Medium population centre (30,000–99,000)	17	23.9		
Small population centre (1,000–29,999)	1	1.4		
Type of certification				
Registered behaviour technician (RBT)	2	2.8		
BCBA	54	76.1		
BCaBA	6	8.4		
BCBA-D	2	21.8		
Other				
BCBA in progress	7			

^a^Respondents were able to select more than one option for these questions.

### 2.2. Measure

As there were no instruments available at time of data collection to measure the construct of interest, a questionnaire was developed with input from diagnosticians and behaviour therapists. The questionnaire included nominal and ordinal data collection, including five-point Likert scales to rate the importance of information, and free text fields to capture any aspects not covered by the presented options. The survey was piloted with three behaviour therapists prior to dissemination. They were asked about survey length, clarity of questions, and whether important content was missing. Based on their feedback, terminology for different types of therapists and training was clarified, as was wording about information obtained by the therapist. Respondents were asked to indicate what diagnostic assessment information they received for new clients, the assessment information they utilise to develop a therapeutic plan (and whether it is obtained in the diagnostic package or obtained by them), the relative importance of these sources of information, and the relative importance of information found in a physician/psychologist assessment. Respondents were asked to rate the importance of 13 diagnostic assessment aspects in order to develop an intervention (Not Important at All, Of Little Importance, Of Average Importance, Very Important, Absolutely Essential). Additionally, respondents were asked to indicate their level of agreement to nine statements related to the quantity, quality, recency, and relevancy of diagnostic information received (Strongly Disagree, Disagree, Neither Agree nor Disagree, Agree, Strongly Agree). Data analysis was completed using means and frequencies.

### 2.3. Procedure

Research ethics approval was obtained from Holland Bloorview Kids Rehabilitation Hospital. Potential participants from the registry were emailed directly, with a total of three emails sent over 4 weeks to recruit respondents. The first page of the survey was related to consent. No identifying information was collected. Responses to the questionnaire are only reported if the survey was fully completed.

## 3. Results

### 3.1. Information received from diagnostic assessments

Respondents receive assessment information from various sources (Table 2). The majority of respondents typically received a brief physician/psychologist report (*n* = 49, 69%), a speech-language pathology (SLP) assessment (*n* = 37, 52.1%), and an individualised education plan (*n* = 36, 50.7%) Respondents were less likely to receive information from diagnostic tools, with the Autism Diagnostic Observation Schedule (ADOS) being the most common (*n* = 16, 22.5%).

**Table 2:** Type of diagnostic assessment information typically received for new clients (*n* = 71).

	Information received *n* (%)	Information utilised for developing plans *n* (%)	
		Provided in diagnostic package	Individually obtained
Type of diagnostic assessment information			
Brief physician/psychologist report	49 (69)	41 (57.7)	6 (8.5)
Speech and language assessment report	37 (52.1)	36 (50.7)	17 (23.9)
Individualised education plan	36 (50.7)	31 (43.7)	18 (25.4)
DSM-5 severity level	35 (49.2)	31 (43.7)	6 (8.5)
Detailed physician/psychologist report	35 (49.2)	32 (45.1)	7 (9.9)
School/daycare report/evaluations	33 (46.5)	30 (42.3)	22 (31)
Psychoeducational assessment report	26 (36.6)	26 (36.6)	6 (8.5)
Occupational therapy assessment report	24 (33.8)	30 (42.3)	14 (19.7)
Results of standardised tools			
ADOS	16 (22.5)		
CARS	6 (8.5)		
ADI-R	3 (4.2)		
Other^a^	12 (16.9)		
Interviews/consultations			
Interview with caregiver		11 (15.5)	65 (91.5)
Consultation with SLP		7 (9.9)	45 (63.4)
Consultation with OT		7 (9.9)	39 (54.9)
Interview with teacher(s)		7 (9.9)	52 (73.2)
Interview with client		6 (8.5)	54 (76.1)
Consultation with psychologist		5 (7)	29 (40.8)
Consultation with other		2 (2.8)	13 (18.3)

^a^Other tools used included: ABLSS, Assessment of Basic Language and Learning Skills; AFLS, Assessment of Functional Living Skills; VB-MAPP, Verbal Behaviour Milestones Assessment and Placement Programme; DP3, Developmental Profile – 3; IQ, intelligence quotient; MAS, Motivation Assessment Scale; FAST, Functional Analysis Screening Tool; QABF, Questions About Behavioural Function.

### 3.2. Elements of the diagnostic assessment that are perceived as important for planning

Respondents were asked to indicate the importance of different sources of information for treatment planning (Table 3). As described, the information that was most commonly received by behaviour therapists was a brief physician/psychologist report. For this type of information, 16.9% (*n* = 12) of respondents indicated it was Absolutely Essential, 38% (*n* = 27) indicated it was Very Important, and 29.6% (*n* = 21) indicated it was Of Average Importance. Information most frequently identified as Absolutely Essential was ‘My own family interview’ (76.1%), ‘My client interview’ (67.6%), and ‘My teacher interview’ (31%). Additionally, consultation with service providers was identified as Very Important by 39.5% of respondents for SLPs, 33.8% for occupational therapists, and 18.3% for physicians.

**Table 3:** Importance of assessments and information for developing an intervention (*n*, %).

	Not important at all	Of little importance	Of average importance	Very important	Absolutely essential
Physician/psychologist assessment report	3 (4.2)	7 (9.9)	21 (29.6)	27 (38)	12 (16.9)
Speech and Language assessment report		7 (9.9)	27 (38)	22 (31)	12 (16.9)
Occupational therapy assessment report	1 (1.4)	14 (19.7)	28 (39.4)	16 (22.5)	7 (9.9)
School/daycare report card, progress report(s), evaluations	1 (1.4)	11 (15.5)	30 (42.3)	19 (26.8)	7 (9.9)
Individual Education Plan	2 (2.8)	15 (21.1)	36 (50.7)	12 (16.9)	4 (5.6)
Psychoeducational assessment report		7 (9.9)	21 (29.6)	26 (36.6)	11 (15.5)
My own (family) interview			1 (1.4)	16 (22.5)	54 (76.1)
My teacher interview		3 (4.2)	16 (22.5)	28 (39.4)	22 (31)
My client interview		1 (1.4)	3 (4.2)	17 (23.9)	48 (67.6)
Consultation with SLP		5 (7)	25 (35.2)	28 (39.4)	8 (11.3)
Consultation with OT	1 (1.4)	8 (11.3)	28 (39.4)	24 (33.8)	6 (8.5)
Consultation with physician	5 (7)	13 (18.3)	28 (39.4)	13 (18.3)	7 (9.9)
Consultation with psychologist	2 (2.8)	10 (14.1)	26 (36.6)	20 (28.2)	9 (12.7)
Information found in physician/psychologist assessment report					
Client demographics	3 (4.2)	17 (23.9)	27 (38)	19 (26.8)	5 (7)
Client’s medical history		5 (7)	18 (25.4)	24 (33.8)	24 (33.8)
Client’s family history	2 (2.8)	8 (11.3)	21 (29.6)	24 (33.8)	16 (22.5)
Client’s social history		3 (4.2)	20 (28.2)	31 (43.7)	17 (23.9)
Client’s gross motor history		3 (4.2)	17 (23.9)	33 (46.5)	18 (25.4)
Client’s fine motor history		3 (4.2)	16 (22.5)	34 (47.9)	17 (23.9)
Client’s adaptive skills history		1 (1.4)	13 (18.3)	32 (45.1)	24 (33.8)
Client’s speech/language history			13 (18.3)	30 (42.3)	27 (38)
Client’s cognitive history				14 (19.7)	27 (38)
Client’s standardised test descriptors					
Quantitative scores	3 (4.2)	15 (21.1)	32 (45.1)	17 (23.9)	4 (5.6)
Qualitative details	3 (4.2)	12 (16.9)	32 (45.1)	19 (26.8)	5 (7)
Services involved		1 (1.4)	15 (21.1)	29 (40.8)	25 (35.2)
Physician/psychologist overall impression		8 (11.3)	30 (42.3)	23 (32.4)	8 (11.3)

### 3.3. Quality, quantity, and relevance of assessment information

Lastly, respondents were asked to indicate their level of agreement with various statements related to the quality, quantity and relevance of the information received and how they use it (Table 4). Most respondents Agreed/Strongly Agreed (74.6%) that information in the physician/psychologist report is often outdated, or older than 6 months. As to whether the type and amount of information received was adequate, responses were more varied. Over 30% of respondents Agreed/Strongly Agreed that the type of information received is adequate, and over 30% Disagreed/Strongly Disagreed with this statement. As well, most respondents Disagreed/Strongly Disagreed that they do not need/use other professional’s assessment when developing an intervention plan. Lastly, responses were also more varied for how the information received from diagnostic assessments impacts the type and quantity of treatments provided. Approximately 45% of respondents Agreed/Strongly Agreed that the information influences the *type* of intervention provided; however, nearly 40% Disagreed/Strongly Disagreed that the information influences *how much* treatment is provided.

**Table 4:** Level of agreement with statements related to quality, quantity, recency, and relevancy of obtained information.

	Strongly Disagree	Disagree	Neither agree nor disagree	Agree	Strongly Agree
Information in the physician/psychologist report is often out-dated (i.e. dated >6 months ago)	1 (1.4)	7 (9.9)	10 (14.1)	12 (16.9)	41 (57.7)
The physician/psychologist report does not include enough detail	4 (5.6)	11 (15.5)	28 (39.4)	19 (26.8)	9 (12.7)
The physician/psychologist report includes too much detail	16 (22.5)	24 (33.8)	28 (39.4)	3 (4.2)	
The type of information I receive is adequate	4 (5.6)	19 (26.8)	26 (36.6)	20 (28.2)	2 (2.8)
The amount of information I receive is adequate	4 (5.6)	13 (18.3)	30 (42.3)	21 (29.6)	2 (2.8)
I do not use other professionals’ assessments when developing an intervention plan	31 (43.7)	18 (25.4)	9 (12.7)	11 (15.5)	2 (2.8)
I do not need other professionals’ assessments when developing an intervention plan	31 (43.7)	24 (33.8)	9 (12.7)	6 (8.5)	1 (1.4)
Information in the diagnostic package influences what type of treatment is provided	5 (7)	14 (19.7)	20 (28.2)	22 (31)	10 (14.1)
Information in the diagnostic package influences how much treatment is provided	9 (12.7)	18 (25.4)	24 (33.8)	14 (19.7)	6 (8.5)

## 4. Discussion

Despite the historical emphasis on the importance of a multidisciplinary autism assessment, there is limited information on how that impacts service delivery and how different professions collaborate. This is the first study to explore what information behaviour therapists receive, their perceived importance of this information, and how it influences service delivery. The main finding of this study is that while behaviour therapists receive diagnostic information from various sources (physicians, SLP, OTs, education), information that is independently obtained (client, caregiver, teacher interview) is relatively more important to their treatment planning.

### 4.1. Implications for collaborative practice

It is unsurprising that many respondents identified the importance of their interview with client/family, as those within the field of ABA have identified family collaboration as a means to achieving family-centred care (13). Autistic children may be engaging in various therapies at the same time, and this work does identify that collaboration with OTs, SLPs, and MDs was viewed as very important by some behaviour therapists. Others have discussed how to achieve collaboration between behavioural analysis, OT, SLP, and psychology (14). They identified limited practice overlap between these professions and called for an enhanced understanding of the contributions of each profession to facilitate collaboration. Exploring what collaboration looks like in practice, understanding how it impacts behaviour therapists’ intervention development, and how other professionals use behaviour therapists’ assessment information would further advance our understanding of how multidisciplinary input impacts service provision.

### 4.2. Implications for diagnostic assessment

Most behaviour therapists identified that the information they receive is outdated, raising issues about requirements at the time of autism diagnostic assessment to guide treatment planning. Given the high demand for autism diagnosis, groups have moved to entrust less specialised assessors, such as general paediatricians, to perform diagnostic assessments (15, 16). Indeed, solo general paediatrician autism assessment is included as an option in the Canadian Paediatric Society autism diagnostic guideline (17). Such models move away from team-based assessments, which were previously deemed necessary to inform therapeutic decisions. Our results challenge this requirement. Diagnostic assessment is often a one-time event, whereas therapeutic decisions will continue throughout the child’s life. Our results show that information about the child can be synthesised from many sources to help inform a therapeutic approach.

### 4.3. Limitations

Our work has some important limitations. A cross-sectional survey does not allow us to understand how informational needs, and the type of information received, have changed over time. There is potential for response bias in that those who responded might feel more strongly about this issue than others. There is also potential for recall bias in thinking about what information is typically received. The survey evaluated perceived importance and relevance; future study could more specifically evaluate what aspects of therapy are influenced by the diagnostic assessment. Most participants indicated they see multiple age groups, and future work can delineate how treatment planning occurs for different age groups. We had limited representation from rural groups; further specific study of therapeutic planning for this group is warranted.

## 5. Conclusion

In summary, behaviour therapists receive and use a variety of diagnostic information in their intervention planning process. They are more reliant on their own assessments to inform intervention planning and delivery, most likely due to receiving out-of-date information. The diagnostic assessment is one tool to inform treatment planning; however, up-to-date information about the child’s needs is likely to be more informative, particularly across the lifespan.

## Data availability statement

The raw data supporting the conclusions of this article will be made available by the authors, without undue reservation.

## Ethics statement

The studies involving humans were approved by Holland Bloorview Research Ethics Board. The studies were conducted in accordance with the local legislation and institutional requirements. The participants provided their written informed consent to participate in this study.

## Author contributions

All authors have participated in the concept and design, analysis and interpretation of data, drafting or revising of the manuscript. All authors contributed to the article and approved the submitted version.

## Funding

This work was supported by Dr. Penner’s Bloorview Children’s Hospital Foundation Chair in Developmental Paediatrics.

## Conflict of interest

MP reports consulting fees from the province of Nova Scotia and Addis & Associates/Roche and research grants from Autism Speaks.

The remaining authors declare that the research was conducted in the absence of any commercial or financial relationships that could be construed as a potential conflict of interest.

## Publisher’s note

All claims expressed in this article are solely those of the authors and do not necessarily represent those of their affiliated organizations, or those of the publisher, the editors and the reviewers. Any product that may be evaluated in this article, or claim that may be made by its manufacturer, is not guaranteed or endorsed by the publisher.

## References

[1] Reichow B Hume K Barton EE Boyd BA. Early intensive behavioral intervention (EIBI) for young children with autism spectrum disorders (ASD). Cochrane Database Syst Rev. (2018) 2018:1–52. doi: 10.1002/14651858.CD009260.pub3 PMC6494600 29742275

[2] Heron TE Cooper JO Heward WL. (2015). Applied behavior analysis. Neukrug ES, editor. United States: The SAGE Encyclopedia of Theory in Counseling and Psychotherapy.

[3] Sandbank M Bottema-Beutel K Crowley S Cassidy M Dunham K Feldman JI. Project AIM: autism intervention meta-analysis for studies of young children. Psychol Bull. (2020) 146:1–29. doi: 10.1037/bul0000215, PMID: 31763860 PMC8783568

[4] Rodgers M Marshall D Simmonds M Le Couteur A Biswas M Wright K. Interventions based on early intensive applied behaviour analysis for autistic children: a systematic review and cost-effectiveness analysis. Health Technol Assess (Winch Eng). (2020) 24:1–306. doi: 10.3310/hta24350, PMID: 32686642 PMC7397479

[5] Ontario Autism Program Advisory Panel. The Ontario autism program advisory panel report recommendations for a new needs-based Ontario autism program. Toronto, ON: King’s Printer for Ontario. (2019). Available at: https://www.publications.gov.on.ca/CL29780

[6] Yingling ME Ruther MH Dubuque EM. Trends in geographic access to board certified behavior analysts among children with autism Spectrum disorder, 2018–2021. J Autism Dev Disord. (2022) 52:5483–90. doi: 10.1007/s10803-021-05402-0, PMID: 34985719 PMC8727480

[7] Dimian AF Symons FJ Wolff JJ. Delay to early intensive Behavioral intervention and educational outcomes for a Medicaid-enrolled cohort of children with autism. J Autism Dev Disord. (2021) 51:1054–66. doi: 10.1007/s10803-020-04586-1, PMID: 32642958 PMC12599662

[8] Government of Ontario. Interprofessional Collaboration. (2019). Available at: https://www.ontario.ca/document/ontario-autism-program-clinical-framework/engagement-and-roles#.

[9] Penner M Anagnostou E Andoni LY Ungar WJ. Systematic review of clinical guidance documents for autism spectrum disorder diagnostic assessment in select regions. Autism. (2018) 22:517–27. doi: 10.1177/1362361316685879, PMID: 28548543 PMC6039866

[10] Zwaigenbaum L Penner M. Autism spectrum disorder: advances in diagnosis and evaluation. BMJ. (2018) 361:k1674. doi: 10.1136/bmj.k1674 29784657

[11] Behavior Analyst Certification Board. Board Certified Behavior Analyst® Handbook. (2023). Available at: https://www.bacb.com/wp-content/uploads/2022/01/BCBAHandbook_230804-a.pdf.

[12] Ontario Association for Behaviour Analysis. Applied Behaviour Analysis Primer. Toronto, ON: ONTABA. (2021). Available at: ontaba.org/wp-content/uploads/2021/07/ABA-Primer.pdf

[13] Brown KR Hurd AM Randall KR Szabo T Mitteer DR. A family-Centered care approach to behavior-analytic assessment and intervention. Behav Anal Pract. (2022). doi: 10.1007/s40617-022-00756-y PMC13083665 42005053

[14] LaFrance DL Weiss MJ Kazemi E Gerenser J Dobres J. Multidisciplinary teaming: enhancing collaboration through increased understanding. Behav Anal Pract. (2019) 12:709–26. doi: 10.1007/s40617-019-00331-y, PMID: 31976281 PMC6743510

[15] McNally Keehn R Swigonski N Enneking B Ryan T Monahan P Martin A. Diagnostic accuracy of primary care clinicians across a statewide system of autism evaluation. Pediatrics. (2023) 152:e2023061188. doi: 10.1542/peds.2023-061188 37461867 PMC10686684

[16] Sohl K Levinstein L James A Greer S Boles K Curran AB. ECHO (extension for community healthcare outcomes) autism STAT: a diagnostic accuracy study of community-based primary care diagnosis of autism Spectrum disorder. J Dev Behav Pediatr. (2023) 44:e177–84. doi: 10.1097/DBP.0000000000001172, PMID: 36978232

[17] Brian JA Zwaigenbaum L Ip A. Canadian paediatric society, autism Spectrum disorder guidelines task force. Standards of diagnostic assessment for autism spectrum disorder. Paediatr Child Health. (2019) 24:444–51. doi: 10.1093/pch/pxz117, PMID: 31660042 PMC6812299

